# Discovery and preclinical efficacy of HSG4112, a synthetic structural analog of glabridin, for the treatment of obesity

**DOI:** 10.1038/s41366-020-00686-1

**Published:** 2020-09-17

**Authors:** Leo Sungwong Choi, In Geun Jo, Ku Suk Kang, Jeong Ho Im, Jiyoung Kim, Jinyoung Kim, Jin Wook Chung, Sang-Ku Yoo

**Affiliations:** 1 Glaceum Inc., Suwon, Republic of Korea; 2 Erum Biotechnologies Inc., Suwon, Republic of Korea

**Keywords:** Drug discovery, Obesity, Metabolic diseases, Energy metabolism, Metabolic syndrome

## Abstract

**Background:**

HSG4112 is a clinical-stage drug candidate for the treatment of obesity. Here, we report its discovery and preclinical efficacy.

**Methods:**

In high-fat diet (HFD)-induced obese male C57BL/6J mice, we tested the weight loss effect of synthetic compounds derived from a structure–activity relationship (SAR) study of glabridin, a natural compound known to reduce body weight and influence energy homeostasis. After selecting HSG4112 as our optimized compound from this discovery method, we characterized its pharmacological effects on parameters related to obesity through in vivo metabolic and biochemical measurements, histology and gene expression analysis, and indirect calorimetry.

**Results:**

Through the SAR study, we identified four novel components of glabridin pertinent for its anti-obesity activity, and found that HSG4112, an optimized structural analog of glabridin, markedly supersedes glabridin in weight reduction efficacy and chemical stability. Six-week administration of HSG4112 to HFD-induced obese mice led to dose-dependent normalization of obesity-related parameters, including body weight, muscle and adipose tissue weight, adipocyte size, and serum leptin/insulin/glucose levels. The weight reduction induced by HSG4112 was partially mediated by decreased food intake and mainly mediated by increased energy expenditure, with no change in physical activity. Accordingly, the pattern of transcriptional changes was aligned with increased energy expenditure in the liver and muscles. Following significant body weight reduction, robust amelioration of histopathology and blood markers of fatty liver were also observed.

**Conclusions:**

Our study demonstrates the key chemical components of glabridin pertinent to its weight loss effects and suggests HSG4112 as a promising novel drug candidate for the pharmacological treatment of obesity.

## Introduction

Obesity occurs when energy intake exceeds energy expenditure. Despite the simplicity of the causal equation, its pharmacological treatment requires a multifaceted approach due to the accompanying burdens of chronic inflammation, hypertension, dyslipidemia, and overall disruption of energy metabolism [1]. Currently, existing FDA-approved anti-obesity drugs are specific receptor-targeted drugs that focus on the initial part of the equation: they reduce energy intake by inhibiting food intake or absorption [2, 3]. Energy intake reduction is equivalent to calorie-restricted dieting and is prone to weight cycling (yo-yo effect) because it decreases muscle mass or muscle activity, leading to reduced metabolic function and energy expenditure [4]; hence, long-term weight loss is difficult to achieve [5, 6]. In consideration of the accompanying hurdles in ameliorating obesity caused by excess energy intake, a promising anti-obesity drug or combination of drugs should consist of multi-functional effects leading to both reduced energy intake and enhanced energy expenditure, with potent clinical efficacy.

Glabridin is a promising base compound in the pharmacological research of multi-functional, energy expenditure-enhancing anti-obesity drugs. A prenylated polyphenolic isoflavan, glabridin is an isolate and the key chemical and biological marker of *Glycyrrhiza glabra* L. (Fabaceae) roots, also commonly known as licorice [7, 8]. Along with a long history of medicinal use of licorice, glabridin has been considered a prospective molecule for ameliorating metabolic diseases [9] due to its anti-oxidative, anti-inflammatory, and anti-atherogenic effects as well as its regulation of energy metabolism [10]. Previously, glabridin in high-fat diet (HFD)-induced obese mice was shown to reduce ~25% body weight, ameliorate lipid dysregulation, and activate the signaling pathway for AMP-activated protein kinase (AMPK) [11], an enzyme well-known for its critical role in energy homeostasis [12].

Glabridin itself has not been developed as a therapeutic drug because of several limitations, but its derivatives show potential. Glabridin has low physicochemical stability [13] and low bioavailability [14]; light, temperature, humidity, and pH all influence the stability of glabridin [13]. In addition, the extraction of glabridin from licorice is complicated and unlucrative because of glabridin’s low content and high decomposition rate in the preparation process [13]. Other compounds isolated from licorice root with glabridin backbone—hispaglabridin A and B and 4′-O-methylglabridin—demonstrated comparable anti-oxidative properties [7], and 3″,4″-dihydro-glabridin showed superior stability and tyrosinase inhibitory effects [15]. Recent studies report glabridin’s putative pharmacophore [10] and the efficient synthesis of racemic glabridin [16], thereby facilitating rational designs and syntheses of glabridin and its derivatives. Yet, the effects of glabridin derivatives as anti-obesity agents and their exact pharmacophore are unknown.

Here, we report the discovery and preclinical efficacy of a novel small oral molecule named HSG4112, which is a synthetic derivative and a structural analog of glabridin currently in clinical trial for obesity. We show that HSG4112, discovered through in vivo phenotypic validation of the structure-activity relationship (SAR) analysis and identification of the pharmacophore for weight loss effects, is a new chemical entity which presents potent preclinical efficacy with an energy expenditure-enhancing effect, and thus is a promising therapeutic drug candidate for the treatment of obesity.

## Materials and methods

### Compounds

HSG4112 and other glabridin-derivative compounds were prepared at Glaceum Inc. (Suwon, Republic of Korea) following the protocols from Patent US9783551B2 [17]. Glabridin was purchased from Sigma-Aldrich Co. (St. Louis, MO, USA).

### Animals and diets

Male C57BL/6J mice were purchased from Jackson Laboratory (Bar Harbor, ME, USA) at 14 weeks of age. The mice were fed high-fat (60% kcal% fat) diets (Research Diets Inc., New Brunswick, NJ, USA) starting at 6 weeks of age or were fed normal diet (10% kcal% fat). Mice were acclimatized for 2 weeks in a controlled environment (12 h light/dark cycle, lights on at 7:00 a.m., 22 ± 2 °C, 54.4 ± 8% humidity, ad libitum access to water and their respective diets) and were randomly divided into groups at 17 weeks of age in a manner in which obese group’s mean body weights were equal. Therefore, the HFD-induced obese group were fed HFD for a total of 11 weeks before the first drug administration. Individual food consumption and body weight were measured weekly unless otherwise noted. Animals in different groups were identified by color-coded cage cards. Animals were dosed via gastric intubation once a day in the afternoon (3:00–4:00 p.m.) for 6 weeks unless otherwise noted. At the end of the study, animals were fasted for 14–16 h and anesthetized by isoflurane inhalation at terminal sacrifice. In the phenotypic screening assays, each group consisted of four animals (*n* = 2 for the vehicle group), with the exception of the enantiomerization step, where each group consisted of five animals. In the main efficacy study, each group consisted of ten animals; one animal from the normal group was excluded due to incidental death (the exclusion criteria were preestablished). The sample sizes were chosen to observe weight-reducing trends of multiple compounds at the screening phase, and to detect sufficient statistical significance in the main study. All animal experiment procedures were in compliance with the Animal Protection Act of Korea and the Guide for the Care and Use of Laboratory Animals. This study was conducted at Biotoxtech Co., Ltd., Republic of Korea (Institutional Animal Care and Use Committees (IACUC) Approval No.: 150289).

### Compound stability analysis

Three sets of 10 mg of HSG4112 and glabridin were each added to 10 ml of 1% HCl in MeOH (v/v%) or 1% NaOH in MeOH (w/v%). At 0, 8, 12, 24, 48, and 72-h time-points, 1 ml aliquots from each condition were placed in HPLC (LC2030C, Shimadzu, Japan) in addition to 9 ml of internal standard (10 mg of (±)−3″,4″-dihydro-4′-O-methyl-glabridin dissolved in 100 ml of acetonitrile). HCl, NaOH, MeOH (Sigma-Aldrich Co.), HPLC-grade acetonitrile and formic acid (Thermo Fisher Scientific, Waltham, MA, USA), and deionized water (Millipore, Bedford, MA, USA) were used. The following HPLC condition was used: mobile phase (A:B = 80:20), A: 0.1 formic acid (MeOH:ACN3 = 1:3), B: 0.1 formic acid (DI water), column (Syncronis C18, 150 × 2.1 mm, 5 µm), flow rate (0.5 ml/min), and UV detection (280 nm). *n* = 3 per condition.

### Histological analysis

Paraformaldehyde-fixed periepididymal fat and liver were paraffin-embedded, sectioned, and stained with Mayer’s hematoxylin-eosin (Sigma-Aldrich Co). The NAFLD activity score (NAS) and fibrosis staging system were applied to liver sections for scoring of steatosis, lobular inflammation, hepatocyte ballooning, and fibrosis as outlined by Kleiner et al. [18]. All histological assessments were performed by a pathologist blind to the treatment.

### Biochemical analysis

Blood samples were collected at sacrifice from the abdominal vein. The following parameters were measured in the serum using a blood-chemical analyzer (7180, HITACHI, Japan): alanine aminotransferase (ALT; JSCC (UV Kinetic)), aspartate aminotransferase (AST; JSCC (UV Kinetic)), total cholesterol (cholesterol oxidase-HMMPS), triglycerides (GPO-HMMPS glycerol blanking), low-density lipoprotein (LDL) cholesterol (selective protection enzymatic), high-density lipoprotein (HDL) cholesterol (direct), and glucose (GHexokinase-G6PDH). Serum insulin level was measured by using the Mouse Insulin ELISA Kit, TBM TMB type (MIT-696, Shibayagi Co., Ltd., Japan). Serum leptin level was measured using the Mouse Leptin ELISA Kit, TMBBM type (MLP-817, Shibayagi Co., Ltd.).

### Quantitative real-time RT-PCR analysis

Total RNA was extracted from the liver, muscle, hypothalamus, and interscapular fat tissues and purified with the RNeasy Mini Kit (Qiagen, Hilden, Germany). cDNA was synthesized using ReverTra Ace qPCR RT Master Mix (Toyobo, Osaka, Japan). The primers for 68 select genes were designed using Primer3; sequences are provided in Supplementary Table 1. *GAPDH* was used for normalization. qRT-PCR was performed using the QuantiSpeed SYBR Green Kit (PhileKorea, Seoul, Republic of Korea). *n* = 4 per group.

### Western blot

HFD-induced obese mice were dosed with 100 mg kg^−1^ of HSG4112 for 11 days prior to sacrifice. The hypothalamus and interscapular adipose tissues were extracted. Western blotting was performed as previously described [19]. Phospho-AMPK alpha 1 (T183) and 2 (T172) and UCP1 (Abcam, Cambridge, United Kingdom) were used as primary antibodies.

### Metabolic analysis

HFD-induced obese mice were given either vehicle only or vehicle with HSG4112 (0.5% feed mixture) for 4 weeks prior to the metabolic rate analysis. Instead of oral administration, HSG4112 was mixed to the diet in order to avoid effects that may occur from taking the animals out of the indirect calorimetry cage for daily dose administration, while maintaining the weight loss effect equivalent to the oral administration of HSG4112 at 100 mg kg^−1^ dose (Supplementary Fig. 1). The mice were placed inside an Oxymax/CLAMS (Columbus Instruments, Columbus, OH, USA) for 24 h prior to the experiment for environmental adaptation, and for an additional 49 h for the experiment. All parameters—VO_2_, VCO_2_, respiratory exchange ratio (RER), energy expenditure, and locomotor activity—were calculated using a built-in software and previously reported method [20]. *n* = 4 per group. This study was conducted at Asan Medical Center, Republic of Korea (approval from the Institutional Animal Care and Use Committee of Asan Institute for Life Sciences).

### Statistical analysis

Statistical analyses were performed using GraphPad Prism 8.3.0 (GraphPad Software Inc., San Diego, CA, USA) by one-way or two-way ANOVA followed by Student’s, Dunnett’s, Tukey’s, or Sidak’s post hoc test as appropriate. A *p* value of <0.5 was considered significant. All density values were quantified using ImageJ.

## Results

### In vivo SAR study of glabridin and its derivatives identifies anti-obesity components of glabridin backbone and the final optimized compound HSG4112

**Fig. 1:**
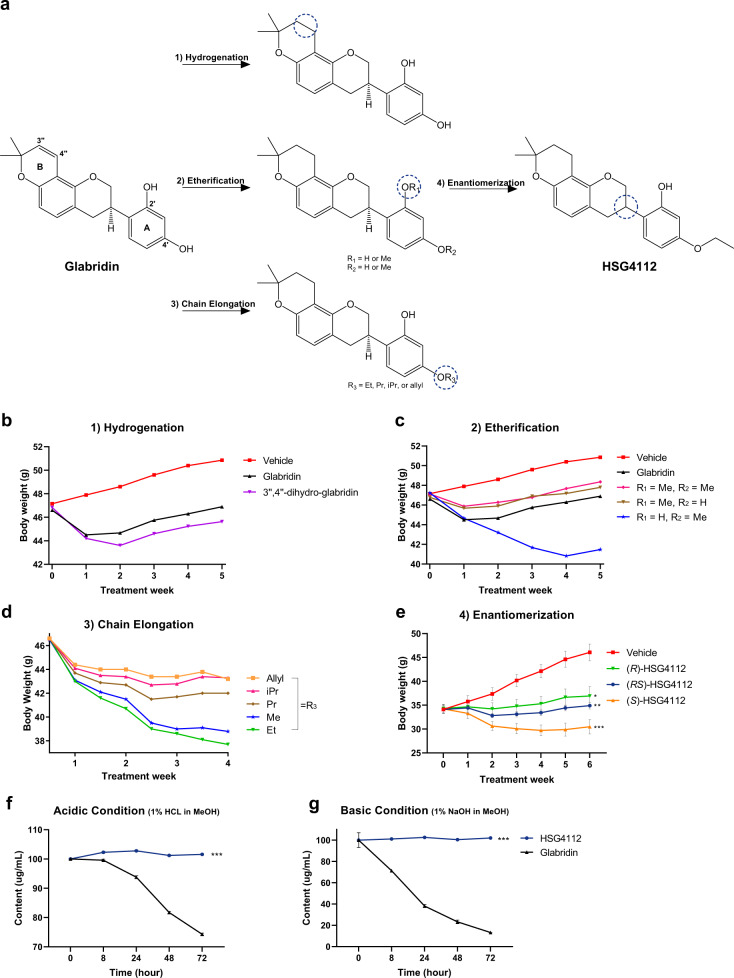
SAR study of glabridin and subsequent structural modifications improve in vivo weight loss effect and chemical stability of the structural analogs. **a** Schematic of the development of HSG4112 from glabridin. HFD-induced obese mice (*n* = 4 for all obese groups and *n* = 2 for the vehicle group) were orally administered with 150 mg kg^−1^ of respective glabridin derivatives for 4–6 weeks in **b** hydrogenation, **c** etherification, and **d** chain elongation steps. Data represent mean only, without statistical analysis, due to low sample size specifically employed for the purpose of screening. **e** HFD-induced obese mice (*n* = 5) were orally administered with 50 mg kg^−1^ of *(R)-, (S)-*, or racemic HSG4112 for 6 weeks for the enantiomerization step. Data represent mean ± SEM. Two-way ANOVA with Dunnett’s multiple comparison test was performed; *p < 0.05, **p < 0.01, ***p < 0.001 vs. vehicle group. Degradation of glabridin and HSG4112 was measured by HPLC in **f** acidic solution (1% HCl in MeOH) and in **g** basic solution (1% NaOH in MeOH). Data represent mean ± SD; error bar is not visible because of negligible deviance. Student’s *t* test was performed; ***p < 0.001 vs. glabridin.

We performed a SAR study to first overcome the chemical instability of glabridin, whose structure is shown in Fig. 1a. Glabridin’s low stability can be attributed to the pyranobenzene structure in ring A, which is labile under acidic conditions or light, and the resorcinol structure shown in ring B, which is labile under basic conditions. Accordingly, we validated the anti-obesity effect of each modified component by orally administering the compounds for 4–6 weeks to male HFD-induced obese C57BL/6J mice fed with HFD for 11 weeks prior to the administration. We used this direct phenotypic screening method instead of in vitro screening to take into account the divergent pathways and multifaceted network of signals needed for ameliorating obesity.

We modified the pyranobenzene structure by hydrogenating the double bond between the carbon atoms at 3″ and 4″ in ring B to create 3″,4″-dihydro-glabridin, using the hydrogenation reaction from the previously reported protocol [15]. 3″,4″-dihydro-glabridin induced greater body weight reduction in HFD-induced obese mice than glabridin (Fig. 1b), demonstrating that the double bond of the pyranobenzene group is not required for the weight-reducing effect of glabridin. Given the improvement in chemical stability and efficacy, we performed hydrogenation for all following synthetic derivatives.

Next, we modified the resorcinol structure while retaining the -oxy backbone of glabridin. We etherified C-2′, C-4′, or both carbons by attaching methoxy groups, using the typical methylation (MeI, K_2_CO_3_ in acetone) process and column separation of the resulting mixture. The etherification allowed us to test whether the hydroxy groups are necessary for the efficacy, and if so, to determine which of them is critical and which of them can be modified to increase stability. We found that hydroxy-to-methoxy modification at C-2′ (R_1_) induced markedly lower weight loss effects than glabridin (Fig. 1c). This suggests that the C-2′ hydroxy group in ring A is a pharmacophore for weight reduction. Surprisingly, while glabridin led to 13.2% weight loss after 5-week administration, hydroxy-to-methoxy modification at C-4′ (R_2_) led to 25.5% weight loss, which is approximately twofold greater in percentage. We report for the first time that the attachment of a methoxy group at C-4′ remarkably improves the weight-reducing action of glabridin.

Given this beneficial effect from hydroxy-to-methoxy modification at C-4′, we tested whether a further chain elongation of the C-4′ alkoxy group would have any differential effects. In terms of weight-reducing efficacy, we found that attaching an ethoxy group at C-4′ surpassed the attachment of methoxy or all other tested alkoxy groups (Fig. 1d). This compound, with optimized chain length, was termed (*R*)-HSG4112 (Hydrogenated Synthetic Glabridin 4112) by the authors. Further lengthening of the alkoxy substituents had diminished yet retained weight-reducing effects.

Lastly, since the chiral syntheses of glabridin derivatives present a serious challenge in methodology and productivity [10], we synthesized racemic HSG4112 and tested its effect on body weight. Glabridin in nature exists in (*R*)-form, and the effect of (*S*)-glabridin and its derivatives is unknown and predictably null; for most cases of small molecular drugs, only one enantiomer is pharmacologically active while the other enantiomer is either inactive or toxic [21, 22]. Surprisingly, we found that at equivalent dose levels, the (*S*)-isomer surpassed both the (*R*)-isomer and racemic HSG4112 in body weight reduction (Fig. 1e). This presents a remarkable discovery of a glabridin derivative with both enantiomers active and with the more potent enantiomer being the unnatural, synthetic (*S*)-form. Given the similar pharmacological effect of both enantiomers and the inefficient and elaborate protocol needed for the synthesis of chiral HSG4112, racemic HSG4112—simply termed HSG4112—was chosen as our most optimized compound.

In order to confirm the increased chemical stability of HSG4112 relative to glabridin, both compounds were placed in acidic and basic conditions in MeOH and their decomposition rates were measured using HPLC (Fig. 1f, g). HSG4112 proved to be dramatically more stable in both conditions. Expectedly, improvement on the pyranobenzene and resorcinol parts of glabridin led to a significant increase in stability.

### HSG4112 fully reverses adiposity in HFD-induced obese mice in a dose-dependent manner

**Fig. 2:**
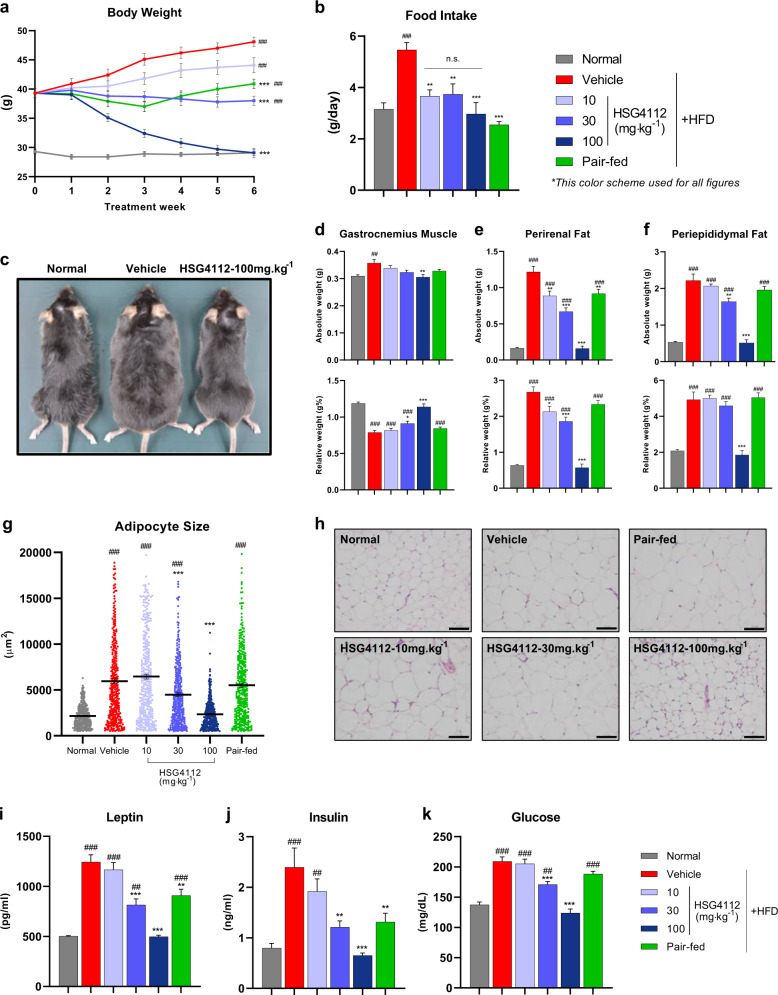
HSG4112 dose-dependently reduces body weight and normalizes obesity-related parameters in HFD-induced obese mice orally administered with 10, 30, or 100 mg kg^−1^ dose for 6 weeks (*n* = 10). Pair-fed group was daily fed at maximum of the food that HSG4112-100 mg kg^−1^ group consumed a day before. **a** Weekly body weights. Data represent mean ± SEM. Two-way ANOVA with Tukey’s multiple comparison test was performed; ^###^*p* < 0.001 vs. normal group and ***p < 0.001 vs. vehicle group. **b** Mean daily food intake. **c** Representative image of mice from normal group, vehicle group, and HSG4112-100 mg kg^−1^ group. Absolute and relative (percentage of gram per body weight gram) weights of **d** gastrocnemius muscle, **e** perirenal fat, and **f** periepididymal fat. **g** Individual white adipocyte size distribution. **h** Representative image of histological section of periepididymal fat stained with H&E. Standard bar = 100 µm. Serum concentration of **i** leptin, **j** insulin, and **k** glucose. For **b**–**k**, all data represent terminal values of mean ± SEM, and one-way ANOVA with Tukey’s multiple comparison test was performed; n.s. not significant, ^##^*p* < 0.05, ^###^*p* < 0.001 vs. normal group and *p < 0.05, **p < 0.01, ***p < 0.001 vs. vehicle group.

After the discovery of HSG4112, we aimed to characterize its full preclinical efficacy in the same experimental setup as the SAR study, using HFD-induced obese mice given different doses of HSG4112 (10, 30, and 100 mg kg^−^^1^) with the addition of a pair-fed group. The pair-fed group was given the amount of feed that HSG4112-100 mg kg^−1^ group consumed the day before. Six-week administration of HSG4112 at 10, 30, and 100 mg kg^−1^ dose led to significant dose-dependent body weight reduction by 4.0 g (8.3%), 10 g (21%), and 19 g (40%), respectively, compared to the 48.1 g body weight of HFD-induced obese mice administered with only the vehicle (hereinafter vehicle group) (Fig. 2a). Expectedly, the plasma concentrations of HSG4112 in the HSG4112-30 mg kg^−1^ and −100 mg kg^−1^ groups at the end of the 6-week administration period showed dose proportionality (Supplementary Fig. 2). At 100 mg kg^−1^ dose, the body weight of HFD-induced obese mice was completely normalized to 29.2 g, equivalent to that of normal chow-fed mice (hereinafter normal group). The body weight of the pair-fed group was 40.9 g; under the assumption that the pair-fed group fully represents the reduction of food intake in the HSG4112-100 mg kg^−1^ group, reduced food intake accounts for 37.8% (−7.2 g) of the weight loss induced by HSG4112 while enhanced energy expenditure accounts for 62.2% (−11.7 g). The mean daily food intake was significantly reduced in all HSG4112-treated groups compared to 5.47 g of daily HFD consumed by the vehicle group (Fig. 2b); HSG4112-10, 30, and 100 mg kg^−1^ groups had 3.67, 3.74, and 2.97 g of daily food intake, respectively. However, no statistically significant difference in food intake reduction was observed between the groups treated with different doses of HSG4112, suggesting incomplete dose proportionality. Representative images from the normal, vehicle, and HSG4112-100 mg kg^−1^ groups are shown in Fig. 2c.

Next, we investigated the effect of HSG4112 on additional parameters of obesity: adipose tissue and muscle mass, adipocyte size, and blood hormone and glucose levels. HSG4112 dose-dependently normalized body weight-relative gastrocnemius muscle weight, while only the HSG4112-100 mg kg^−1^ group showed significant decrease in absolute gastrocnemius muscle weight (Fig. 2d). Significant and dose-dependent reduction of both relative and absolute white adipose tissue (WAT) masses in abdominal fat—periepididymal and perirenal adipose tissues—were observed (Fig. 2e, f) in addition to the similar degree of effects in individual adipocyte size (Fig. 2g, h). Additionally, leptin and insulin are key hormones in mediating glucose homeostasis, and their blood levels are indicative markers of adiposity and negative predictors for future weight gain [23, 24]. HSG4112 significantly decreased serum leptin, insulin, and glucose concentration to a normal level (Fig. 2i–k) in a dose-dependent manner. In summary, dose-dependent amelioration of adiposity and full normalization of adiposity in the HSG4112-100 mg kg^−1^ group were observed. Given the immense beneficial effect of HSG4112 at 100 mg kg^−1^, this dosage was used for treatment groups in the following analyses (hereinafter HSG4112 group).

### HSG4112 markedly enhances energy expenditure in HFD-induced obese mice

**Fig. 3:**
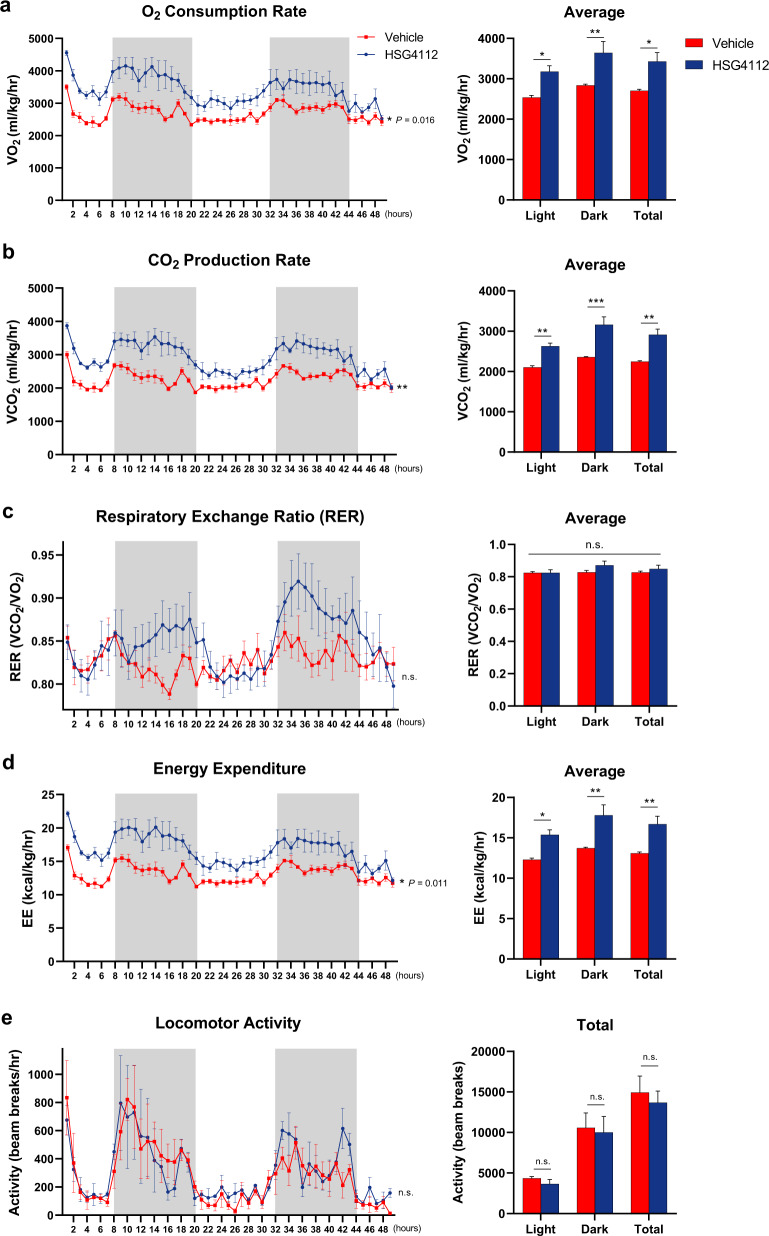
HSG4112 enhances energy expenditure in HFD-induced obese mice. For assessing in vivo energy expenditure, HFD-induced obese mice that were fed HSG4112 (0.5% feed mixture) for 4 weeks were placed in an Oxymax/CLAMS device (*n* = 4). **c** Oxygen consumption rate, **d** carbon dioxide production rate, **e** respiratory exchange ratio, and **f** energy expenditure were measured and normalized to body weight. **g** Locomotor activity was measured by number of beam breaks per hour. Data represent mean ± SEM. Two-way ANOVA with Sidak’s multiple comparison test was performed; *p < 0.05, **p < 0.01, ***p < 0.001 vs. vehicle group. Gray area represents dark hours, starting at 8 p.m.

In order to confirm in vivo energy expenditure-enhancing effect of HSG4112, we placed HFD-induced obese mice treated with HSG4112 or vehicle for 4 weeks into open-circuit indirect calorimetry cages [25] to measure respiration, movement, and energy consumption for two consecutive days. HSG4112 significantly increased the overall oxygen consumption rate and carbon dioxide production rate during both light and dark hours (Fig. 3a, b). The respiratory exchange ratio (RER), which is an indicator of predominant substrate utilization for energy usage as either carbohydrate or fat, did not change significantly (Fig. 3c). HSG4112 also significantly increased the overall energy expenditure (Fig. 3d) in both light and dark hours—physically active and inactive hours—without increasing physical activity (Fig. 3e), suggesting increased basal metabolism rate. Dorsal surface temperature, measured by infrared thermography, was not affected by HSG4112 administration (data not shown). Overall, the above results show that HSG4112 increases energy expenditure in vivo.

### HSG4112 regulates metabolic gene expression toward increased energy expenditure in liver and muscles

**Fig. 4:**
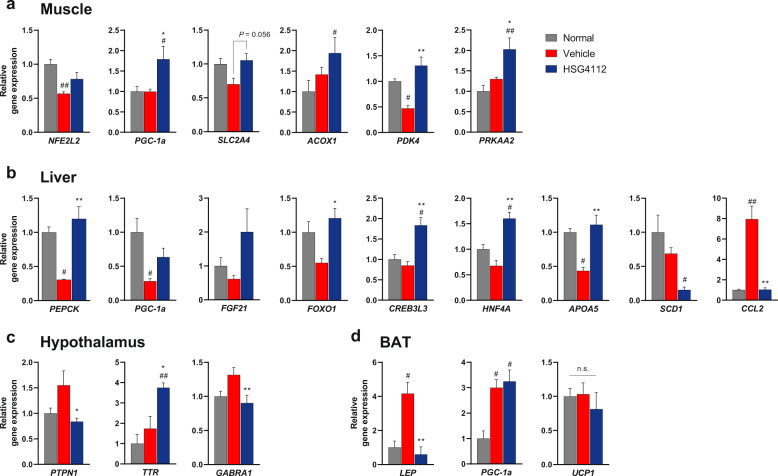
HSG4112 regulates metabolic gene expression in HFD-induced obese mice. Relative gene expression was measured using qRT-PCR (*n* = 4) on terminal **a** gastrocnemius muscle, **b** liver, **c** hypothalamus, and **d** interscapular adipose tissue (BAT) collected from HFD-induced obese mice orally administered with 100 mg kg^−1^ dose for 6 weeks. One-way ANOVA with Tukey’s multiple comparison test was performed; *p < 0.05, **p < 0.01 vs. vehicle group, ^#^*p* < 0.05, ^##^*p* < 0.01 vs. normal group.

To further gauge the effects of HSG4112 on obesity, we investigated the expression levels of genes related to energy metabolism, leptin and insulin signaling, and inflammation. A total of 68 genes were selected through literary search [26–28], and we performed qRT-PCR on mRNA extracted from the hypothalamus, liver, gastrocnemius muscle, and interscapular tissue (BAT) of the HSG4112 group at terminal sacrifice after 6 weeks of treatment. All genes with significant difference in their expression level are shown in Fig. 4a–d; their list and primer sequences are available in Supplementary Table 1.

Amongst the muscle, liver, hypothalamus, and BAT, expressions of the selected genes were most affected by HSG4112 in the muscles and liver. In muscles, genes related to fatty acid oxidation (*ACOX1*, *PRKAA2*) were significantly upregulated, while genes related to glucose transport and metabolism (*SLC2A4*, *PDK4*) were normalized after HSG4112 treatment (Fig. 4a). In the liver, genes related to glucose metabolism (*FGF21*, *PEPCK*), insulin signaling (*FOXO1*, *HNFA4*), and lipid metabolism (*CREB3L3*, *APOA5*, *SCD1*) were induced by HSG4112 to either be normalized or increase toward the direction of increased energy expenditure (Fig. 4b). Relatively few changes were observed in the hypothalamus and BAT. In the hypothalamus, metabolism enhancing *TTR* expression was upregulated, and the *PTPN1* level denoting insulin resistance was downregulated (Fig. 4c), while *NPY*, *AGRP*, *POMC*, *CART* levels denoting leptin resistance showed a normalizing trend (Supplementary Fig. 3). In BAT, the *LEP* level was robustly normalized (Fig. 4d). The mRNA level of *UCP1*, which is one of the proteins well-known for enhancing energy expenditure through futile cycling of mitochondrial potential and consequent thermogenesis in BAT [29], was not influenced by HSG4112 in all tissues. *PGC-1ɑ*, the master regulator of mitochondrial biogenesis [30], was consistently upregulated in the BAT, liver, and muscles, suggesting a possible role of this gene or its pathway in the mechanism of action of HSG4112. Inflammation-related genes (*CCL2*, *NFE2L2*) also had significant change in expression in the liver and muscles.

The majority of the affected genes suggested enhancement of energy expenditure as HSG4112’s mode of action. Therefore, we further investigated whether HSG4112 acts through two well-known enhancers of energy expenditure—AMPK and UCP1—by measuring their protein levels at an early timepoint of the 11-day administration, when body weight just begins to decrease. We found that phosphorylated hypothalamic AMPK markedly decreased to approximately half-fold in the HSG4112 group (Supplementary Fig. 4); decrease in hypothalamic phospho-AMPK signifies activation of peripheral AMPK signaling and increased energy metabolism [31]. Consistent with the transcriptome data, HSG4112 had no effect on the UCP1 protein level in BAT (Supplementary Fig. 4), confirming that HSG4112’s mode of action does not converge with UCP1 activation.

### HSG4112 ameliorates features of fatty liver in HFD-induced obese mice

After observing the robust effect of HSG4112 on adiposity, we examined parameters associated with fatty liver, such as liver weight, liver histology, and blood markers of liver injury. This approach was based on the knowledge that obesity is often accompanied by and causal to the development of nonalcoholic fatty liver disease (NAFLD) and nonalcoholic steatohepatitis (NASH), whose prevalence is dramatically growing worldwide without any approved pharmaco-therapeutic drug up to the current date. Fatty liver disease is characterized by varying degrees of hepatic steatosis, lobular inflammation, cell ballooning, and fibrosis [32].

**Fig. 5:**
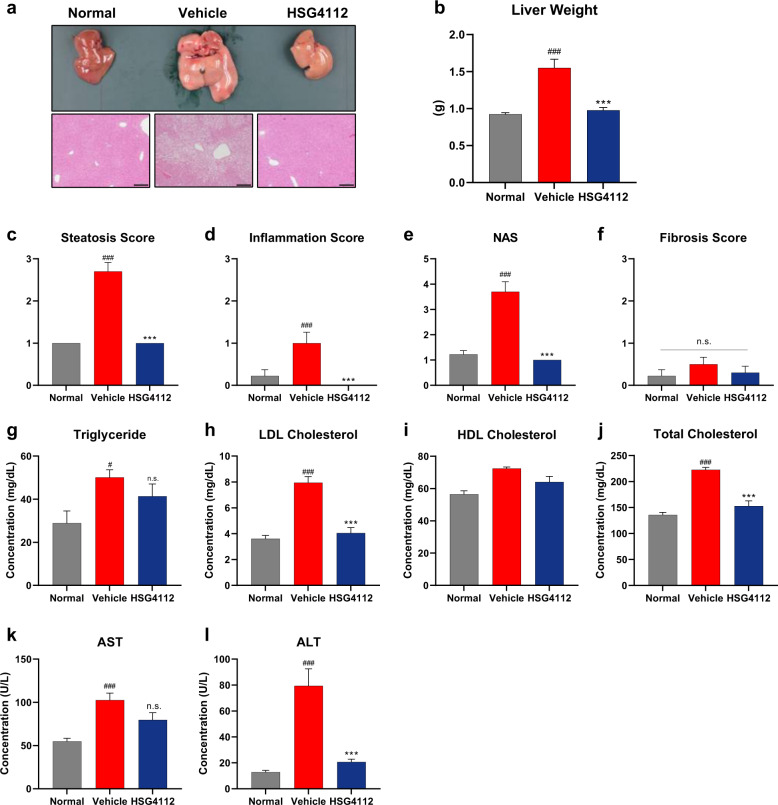
HSG4112 ameliorates fatty liver in HFD-induced obese mice orally administered with 100 mg kg^−^^1^ dose for 6 weeks (*n* = 10). **a** Representative image of liver and its histological section stained with H&E. Standard bar = 200 µm. **b** Quantification of liver weight. NAS and fibrosis score on liver histology: **c** steatosis score, **d** lobular inflammation score, **e** NAS score, and **f** fibrosis score. Serum parameters associated with fatty liver: **g** triglyceride, **h** LDL cholesterol, **i** HDL cholesterol, **j** total cholesterol, **k** AST, and **l** ALT. All data represent terminal values of mean ± SEM. One-way ANOVA with Tukey’s multiple comparison test was performed; n.s. not significant, ^#^*p* < 0.05, ^###^*p* < 0.001 vs. normal group, ***p < 0.001 vs. vehicle group.

At 100 mg kg^−1^ dose, HSG4112 normalized the absolute liver weight and histology in HFD-induced obese mice (Fig. 5a, b). In terms of the NAFLD activity score (NAS), HSG4112 fully reduced the steatosis and inflammation score, and the concurrent overall NAS of HFD-induced obese mice, down to the normal group’s level (Fig. 5c–e). Fibrosis was not significantly induced in the vehicle group (Fig. 5f), and hepatocyte ballooning was not induced at all in all groups (data not shown). Together, HSG4112 improved liver histology mainly through the resolution of inflammation and steatosis.

In terms of blood parameters, treatment of HSG4112 led to reducing trends in serum triglyceride (Fig. 5g) and HDL cholesterol levels (Fig. 5i), and significantly reduced and normalized LDL cholesterol (Fig. 5h) and total cholesterol levels (Fig. 5j); serum triglyceride and HDL cholesterol levels in murine models are challenging to interpret and to apply to fatty liver status [33]. The serum AST level showed a decreasing trend (Fig. 5k) and the ALT level was significantly reduced (Fig. 5l). In sum, HSG4112 normalized most of the relevant blood parameters, indicating robust amelioration of fatty liver in HFD-induced obese mice.

## Discussion

In this study, three main findings arose. First, four structural modifications to glabridin—3″,4″ double bond hydrogenation, C-2′ hydroxy group preservation, C-4′ etherification, and enantiomerization—enhanced anti-obesity efficacy and chemical stability. Second, the optimized analog HSG4112 displayed robust dose-dependent effects on adiposity and fatty liver in HFD-induced obese mice. Third, HSG4112’s mode of action appears to be partially reduction in food intake and mainly increase in energy expenditure. These findings contribute to understanding the structure–activity relationship of glabridin and its anti-obesity effects, and of HSG4112’s preclinical efficacy on obesity and its potential mode of action.

HSG4112 as a synthetic analog superseded glabridin and is a distinctive new chemical entity. Evidence exists in literature to explain its superiority. Dehydrogenation at the C-3″,4″ double bond could have increased its activity because the consequent structural flexibility allows the resorcinol group to interact with the compound’s target or targets more effectively [15]. Ethoxylation at the 4′ carbon, as Bae et al. suggests, may have improved drug bioavailability and stability within the body; metabolic clearance likely happens through the glucuronidation of the C-4′ hydroxy group in glabridin [34], which is absent in HSG4112. Still, the exact biochemical mechanisms of the four modified components are unknown. Examining the unanswered questions of our SAR study may provide additional drug compounds and aid in understanding the molecular mechanisms of glabridin and HSG4112.

The anti-obesity effect of HSG4112 in HFD-induced obese mice is striking, especially at 100 mg kg^−1^ dose, where all examined parameters were fully normalized. Increased energy expenditure appears to be the main mode of action of HSG4112, while appetite control also plays a notable role. The mean daily food intake was significantly reduced starting from HSG4112-10 mg kg^−1^ group but was not further significantly reduced in HSG4112-30 mg kg^−1^ and 100 mg kg^−1^ groups. Dose-dependency was not as clearly observed in food intake as other obesity-related parameters, suggesting that appetite control is not the main effect of the drug, or that appetite control and energy expenditure occur through two different mechanisms where the former reaches maximum efficacy at a low-dose level and the latter increases dose-dependently in efficacy up to the high-dose level. HSG4112-30 mg kg^−1^ had almost equivalent reduction of food intake as HSG4112-10 mg kg^−1^ but noticeably greater effect on body weight, fat mass and adipocyte size, and serum markers of obesity; therefore, the energy expenditure-enhancing effect seems to be at play starting at this dosage, and indirect calorimetry data at this dosage would further support this hypothesis. While muscle is mainly responsible for expending energy, only gastrocnemius muscle mass was measured in this experiment; a more comprehensive analysis of the body composition of lean and fat mass through methods like dual-energy X-ray absorptiometry scan [35] will be of great benefit.

As qRT-PCR was performed on 68 select genes, unbiased RNAseq at an early timepoint before weight reduction could further benefit transcriptomic analysis and interpretation of primary or causal signals induced by HSG4112. Furthermore, while phosphorylated hypothalamic AMPK levels were tested, AMPK levels in peripheral tissues—specifically liver, muscle, and fat—and in respective cells should be measured for further confirmation of the activation of AMPK signaling. Additionally, transcriptomic analysis on WAT would improve interpreting signals or changes related to energy metabolism, leptin signaling, and inflammation. Still, within our current approach, we observed consistent gene regulation patterns in the direction toward enhanced energy expenditure in the liver and muscles: upregulation of fatty acid oxidation, lipid metabolism, and glucose metabolism. This is consistent with signals induced by exercise; the observed increase in *PDK4* and *PGC-1ɑ* levels in muscle tissue similarly occurs after exercising [36, 37]. Because *PGC-1ɑ* regulates mitochondrial biogenesis and AMPK is known to mediate mitochondrial fission [38], HSG4112 may act to improve mitochondrial function or dynamics. On the other hand, evidence of unaffected UCP1 mRNA and protein levels as well as unchanged body temperature in animals suggest that HSG4112 increases energy expenditure in a UCP1-independent manner. It remains unknown whether the above transcriptomic changes are primary or secondary, and such investigation merits attention.

The molecular targets of HSG4112 have not been unraveled. However, along with the assumption that the C-4′ hydroxy group delays metabolic clearance and the C-2′ hydroxy group is the active pharmacophore for both HSG4112 and glabridin, putative targets of HSG4112 may coincide with known or putative targets of glabridin. One potential target is the peroxisome proliferator-activated receptor gamma (PPAR-γ) protein, which is considered the master regulator of adipogenesis, is involved in macrophage inflammatory response, and has been a major target for the pharmacological treatment of type 2 diabetes (T2D) and obesity [39, 40]. Glabridin has been reported to show significant PPAR-γ-binding activity [41] and to upregulate the PPAR-γ mRNA level in HFD-induced obese mice after 8-week administration [42]. Another potential direct target is the paraoxonase 1 (PON1) protein, which is an anti-atherogenic enzyme forming part of the circulating HDL; glabridin was shown to directly interact with recombinant PON1 to reduce linoleic acid-induced oxidation [43]. Potential targets for HSG4112’s appetite control effect remain elusive; glabridin is reported to have in vivo neuroprotective effects of elevating antioxidants—superoxide dismutase and reduced glutathione—and inhibiting effects on staurosporine-induced apoptosis in vitro [44], but the molecular target mediating these neurological effects is unknown. One must note that HSG4112 may have novel or distinct targets compared to glabridin, given its structural modifications and distinctive weight loss effects. The target deconvolution of small molecules discovered through phenotypic screening remains a considerably significant challenge [45]. Novel techniques, such as mass spectrometry-based cellular thermal shift assay (CETSA), where the drug’s binding to proteins in a cell or cell lysate is gauged by the degree of their thermal shifts [46], will be a comprehensive and unbiased method useful in identifying the molecular target or targets of HSG4112.

As HSG4112 showed robust preclinical efficacy on obesity, thus NASH and T2D are ideal secondary target indications. Notable results in liver histology and normalization of parameters related to glucose imbalance [23] support HSG4112’s development for NASH and T2D, respectively. Furthermore, weight loss itself is immensely beneficial to NASH [47] and T2D [48]. As the body weight of the HSG4112 group was fully normalized after 6-week administration, it is unclear whether all the beneficial effects are primary or secondary to weight loss; therefore, future investigation into the primary target cells for these diseases is needed. Of note, despite improvement in histological steatosis score, the liver color of the HSG4112 group was similar to that of the vehicle group, and serum triglyceride levels did not fully normalize; this suggests that ameliorating steatosis may not be HSG4112’s primary effect on the liver. Additionally, hepatocyte ballooning and fibrosis were absent in a HFD-induced obese mouse model, and liver biopsy was not done pre-to-post to account for the variability of NASH histology [49]; supplementary liver fibrosis animal models [50] or liver-specific fat accumulation model [51] will be of value in determining HSG4112’s prospect for treating NASH.

Overall, our results show that HSG4112 is a novel compound derived from glabridin, with potent preclinical efficacy and both energy expenditure-enhancing and appetite-controlling effects. A key factor that enabled this discovery is in vivo phenotypic screening, which takes into account the holistic aspect of biological pathways, is more human-translatable, and applicable toward other relevant indications. Due to this method of approach, the exact mechanism of HSG4112 remains unknown and potentially novel; investigation into such mechanism will provide a meaningful understanding of energy metabolism and enable further development of drugs targeting metabolic diseases. Currently, HSG4112 is in phase 1 clinical trial for obesity. Human translation and proof of concept will demonstrate the potent effect of HSG4112 in treating metabolic diseases.

## Supplementary information

Supplementary Material
