# Supplementary material for: Discovery and preclinical efficacy of HSG4112, a synthetic structural analog of glabridin, for the treatment of obesity
Source: Int J Obes (Lond). 2020 Sep 17;45(1):130–42. doi: 10.1038/s41366-020-00686-1 (PMC7752758; doi:10.1038/s41366-020-00686-1)
Supplement: Supplementary file 1 — Supplementary Material [file 41366_2020_686_MOESM1_ESM.docx]

**Supplementary Information**

*Methods*

**Plasma Concentration**

In addition to animals from the main efficacy study, a total of 18 male C57BL/6J mice (9 per 30 and 100 mg·kg^-1^ dose groups) were purchased, housed, and treated as written in ‘Animals and diets’ section in *Materials and Methods*. Blood samples were collected at Week 0, 3, and 6 of the drug administration at time points of 0, 0.5, 1, 2, 4, 6, 8, 12, 24 hours, which were pooled from three sets of three animals (set 1: 0.5, 4, 12hr; set 2: 1, 6, 24hr; set 3: 2, 8hr) in order to minimize the side effects of blood sampling on mice. Blood samples (0.1 ml) were collected from the cephalic vein. 50 µL of plasma was treated with 150 µL of internal standard (HSG4112-d5, where five hydrogens at C-4’ ethyl group are replaced to five deuterium; Glaceum Inc., Suwon, Republic of Korea), and was analyzed for HSG4112 concentration using LC-MS/MS analysis. HPLC grade methanol (MeOH), acetonitrile (ACN), acetic acid (J.T. Baker, Phillipsburg, NJ, USA), and deionized water (DW) (Millipore, Bedford, MA, USA) were used. LC condition: mobile phase (A:B = 30:70) A: 0.1% acetic acid in DW B: 0.1% acetic acid in (MeOH:ACN = 1:3), column (Kinetex 2.6µm C18 100A, 2.1 x 50mm, Phenomenex Inc.), flow rate (0.2ml/min), injection volume (2 µL). MS condition: capillary voltage (3.0 kV, negative mode), cone (50V), collision energy (23V), source temperature (120°C), desolvation temperature (350°C), desolvation gas flow (650 L/hr), cone gas flow (15 L/hr), precursor to production ion (HSG4112 353.3-> 137.0 m/z, internal standard = 358.3-> 142.0).

**Supplementary Table 1. List of genes subjected to qRT-PCR and their sequences**

| **No.** | **Gene** | **Primer sequences (5′-3′)** | | **Size (bp)** |
| --- | --- | --- | --- | --- |
|  |  | **Forward** | **Reverse** |  |
| 1 | *Ptpn1* | CGGCTATTTACCAGGACATTC | TGCGGTTGAGCATGACCAC | 288 |
| 2 | *LEP* | CTGGAAGCCTCACTCTACTCC | ACATGATTCTTGGGAGCCTG | 80 |
| 3 | *ObRa* | GAAGTCTCTCATGACCACTACAGAT | TTGTTTCCCTCCATCAAAATGTAA | 98 |
| 4 | *ObRb* | GCATGCAGAATCAGTGATATTTGG | CAAGCTGTATCGACACTGATTTCTTC | 81 |
| 5 | *Ghrl* | ACAAGTAACCACGGACAGGC | TGGTAGGAGAGTGCTGGGAG | 102 |
| 6 | *Npy* | CAGAAAACGCCCCCAGAA | AAAAGTCGGGAGAACAAGTTTCATT | 77 |
| 7 | *Agrp* | CGGAGGTGCTAGATCCACAGA | AGGACTCGTGCAGCCTTACAC | 69 |
| 8 | *Pomc* | CATTAGGCTTGGAGCAGGTC | GTCTGGCTCTTCTCGGAGGT | 169 |
| 9 | *Cartpt* | CGAGAAGAACTACGGCCAAG | TCCCTTCACAAGCACTTCAA | 141 |
| 10 | *PRKAA1* | TGCTACTCCACAGAGATCGG | GTCTGAGGGCTTTCCTTGAG | 91 |
| 11 | *PRKAA2* | AACACAACGAAGCCCAAGTC | GCTCGGTACACTTCAGCCAT | 104 |
| 12 | *Sirt1* | CGGCTACCGAGGTCCATATAC | ACAATCTGCCACAGCGTCAT | 135 |
| 13 | *BMP8B* | GGAGCCACACTGGAAGGAAT | CCTGTTGGAGTGCTCTTGGA | 163 |
| 14 | *PPARGC1A* | AGAAGCGGGAGTCTGAAAGG | CAGTTCTGTCCGCGTTGTG | 116 |
| 15 | *Socs3* | AGAAGATTCCGCTGGTACTG | GCTGGGTCACTTTCTCATAGG | 114 |
| 16 | *Prkcq* | CCAGTGCCGACAGTGTAATG | GGAACATGGTTTCTCGGCTA | 200 |
| 17 | *Pik3r1* | GCTCCTGGAAGCCATTGAGAA | CGTCGATCATCTCCAAGTCCA | 134 |
| 18 | *SCD1* | TGGGTTGGCTGCTTGTG | GCGTGGGCAGGATGAAG | 150 |
| 19 | *CIDEC* | AGCTAGCCCTTTCCCAGAAG | GGCAGCCAATAAAGTCCTGA | 96 |
| 20 | *CYCS* | GGATTCTCTTACACAGATGCCA | GTCTGCCCTTTCTCCCTTCT | 147 |
| 21 | *Foxo1* | CAAAGTACACATACGGCCAATCC | CGTAACTTGATTTGCTGTCCTGAA | 84 |
| 22 | *Foxo3* | AACAGACCAGCCACCTTCTCTT | TGAAGCAAGCAGGTCTTGGA | 69 |
| 23 | *Akt2* | GAGGACCTTCCATGTAGACT | CTCAGATGTGGAAGAGTCAC | 148 |
| 24 | *Pdk1* | CCGGGCCAGGTGGACTTC | GCAACTCTTGTCGCAGAAACATAAA | 124 |
| 25 | *Irs2* | GTCCAGGCACTGGAGCTTT | GCTGGTAGCGCTTCACTCTT | 107 |
| 26 | *Bcl2* | TGAGTACCTGAACCGGCATCT | GCATCCCAGCCTCCGTTAT | 57 |
| 27 | *Pdx1* | GAGGACCCGTACTGCCTACA | TTCAACATCACTGCCAGCTC | 111 |
| 28 | *Bax* | CTGCAGAGGATGATTGCTGA | GATCAGCTCGGGCACTTTAG | 174 |
| 29 | *Map3k5* | GACAACGAACAGACGATTGG | TGTCACCCTTTATATCCCGGT | 100 |
| 30 | *Kng1* | GAAGAAGCGCAGGAAATTGA | CGATCACTCGGTGCAACATA | 121 |
| 31 | *Apoa1* | TGTGTCCCAGTTTGAATCCTC | TCATCTCCTGTCTCACCCAAT | 173 |
| 32 | *Apoa5* | GAACGCTTGGTGACTGGAAT | TCGCCTTACGTGTGAGTTTG | 133 |
| 33 | *Pepck* | CCCTGGGAGATGGGGAGTTC | CCCACCATATCCGCTTCCAA | 164 |
| 34 | *Slc2a4* | TCCTTCTATTTGCCGTCCTC | GTTCTGTACTGGGTTTCACCTC | 150 |
| 35 | *Rbp4* | CATCGACACGGACTACGACA | GGCCTGCTTTGACAGTAACC | 216 |
| 36 | *FNDC5* | TCATTGTTGTGGTCCTCTTC | GCTCGTTGTCCTTGATGATA | 81 |
| 37 | *UCP1* | AATGACTGGAGGTGTGGCAG | TGGTGGCTATAACTCTGTAAGCATT | 137 |
| 38 | *UCP2* | CAGCGCCAGATGAGCTTT | GTATCTCCGACCACCACCAG | 210 |
| 39 | *UCP3* | TGGTAAAGACCCGATACATGAAC | CATCATCACGTTCCAAGCTC | 152 |
| 40 | *FASN* | CCCGGAGTCGCTTGAGTA | GGATTTGGTGGAGCCAATTA | 133 |
| 41 | *CPT1A* | CTGCATTCCTTCCCATTTGA | AACTTGCCCATGTCCTTGTAA | 119 |
| 42 | *SREBF1* | ATGCTCCAGCTCATCAACAA | CACGGACGGGTACATCTTTA | 240 |
| 43 | *PPARA* | AGTTCGGGAACAAGACGTTG | CAGTGGGGAGAGAGGACAGA | 110 |
| 44 | *ACOX1* | TTCCAATCATGCGATAGTCC | CGGTAATTGTCCATCTTCAGGTA | 189 |
| 45 | *NRF1* | ACAGGGAAGAAACGGAAACG | ACCACATTCTCCAAAGGTGC | 209 |
| 46 | *TNF* | ACGGCATGGATCTCAAAGAC | GGAGGTTGACTTTCTCCTGGT | 162 |
| 47 | *IL6* | AACGATGATGCACTTGCAGA | GAGCATTGGAAATTGGGGTA | 283 |
| 48 | *CCL2* | CAAGAAGGAATGGGTCCAGA | AAGTGCTTGAGGTGGTTGTG | 182 |
| 49 | *SERPINE1* | CTCTCCGCCCTCACCAAC | GCCAGGGTTGCACTAAACAT | 173 |
| 50 | *iNOS* | GCATCCCAAGTACGAGTGGT | TGTTGTAGCGCTGTGTGTCA | 176 |
| 51 | *CCKAR* | CCAACCTGCTCAAGGATTTC | AAGCATGGGACTTTGTTTGC | 174 |
| 52 | *CCKBR* | GGAAGACAGTGATGGCTGC | CTTCTTAGCCAGCAGCTTGG | 127 |
| 53 | *GABRA1* | TGTACACCATGAGGTTGACCGT | GAAGTCTTCCAAGTGCATTGGG | 56 |
| 54 | *MTNR1A* | AGTGGCTGTTCGTGGCTAGT | GGGTATTAGTGGAGAGGGCTT | 136 |
| 55 | *MTNR1B* | CCTAGTTGGTCTGGGTCAGC | AACAAATTACCTGCGTTCCG | 176 |
| 56 | *SLC6A2* | ATGACATCGGCCTGCCTC | GGCTGTGATCCAGACAACCT | 158 |
| 57 | *SLC6A3* | AGTGGAGGTTCAAGAGCGG | CCCGGCAATAACCATGAA | 169 |
| 58 | *SLC6A4* | TGGGTTTGGATAGTACGTTCG | GATGTCAGTGTGAGCAGGGA | 148 |
| 59 | *MAPK1* | TCTCCCGCACAAAAATAAGG | TCGTCCAACTCCATGTCAAA | 213 |
| 60 | *PTEN* | CAATCATGTTGCAGCAATTCACT | CCCCATAAAAATCTAGGGCCTCT | 122 |
| 61 | *PENK* | GCCTTGTCAATGATGTTTCTTGTC | AACATAGCCATAAGAGACCAATACTG | 150 |
| 62 | *NFE2L2* | CTTGGGCCACTTAAAAGACGAG | TTGCCATCTCTGGTTTGCTG | 186 |
| 63 | *FGF21* | AGGGAGGATGGAACAGTGGT | CAGCAGCAGTTCTCTGAAGC | 195 |
| 64 | *SLC2A1* | ATCCACCACACTCACCACG | CCAACAGGTTCATCATCAGC | 137 |
| 65 | *PDK4* | TGACTCAAAGACGGGAAACC | TGTGGTGAAGGTGTGAAGGA | 209 |
| 66 | *CREB3L3* | TGGACAACCTGACAGAAGAGC | TGAGCAGTGGTTCTGAAGCC | 111 |
| 67 | *HNF4A* | GATTGCCAACATCACAGACG | ATCCAGAAGGAGTTCGCAGA | 103 |
| 68 | *TTR* | ATGGCTTCCCTTCGACTCTT | GCATCCAGGACTTTGACCAT | 116 |


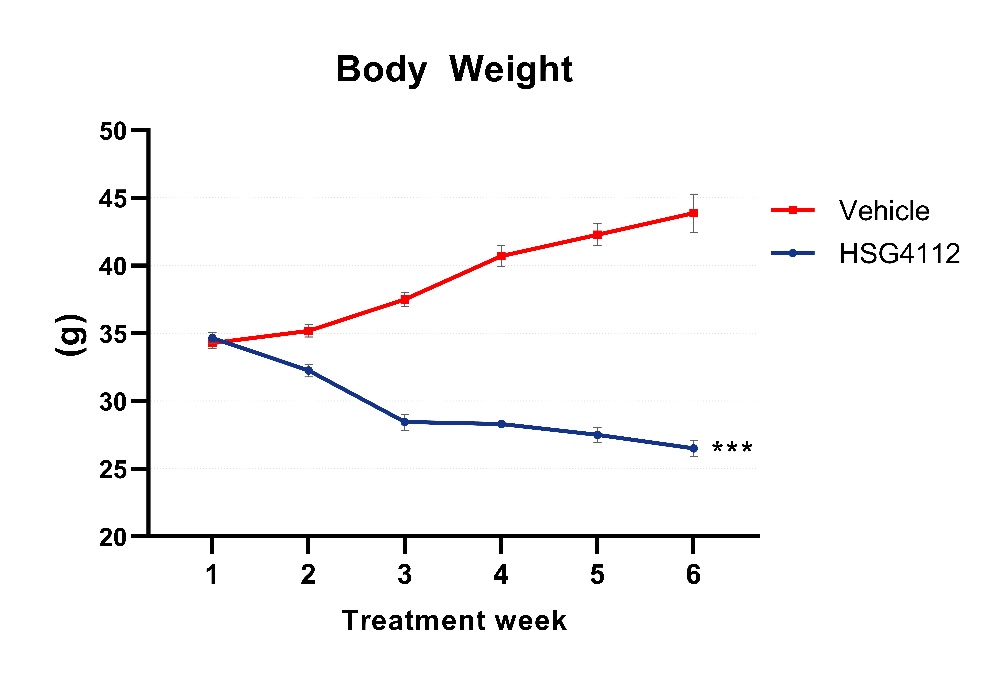


**Supplementary Fig. 1 Body weight of HFD-induced obese mice (n = 5) used for the CLAMS metabolic assay. Mean ± SEM. Student’s t-test. ****P* < 0.001**


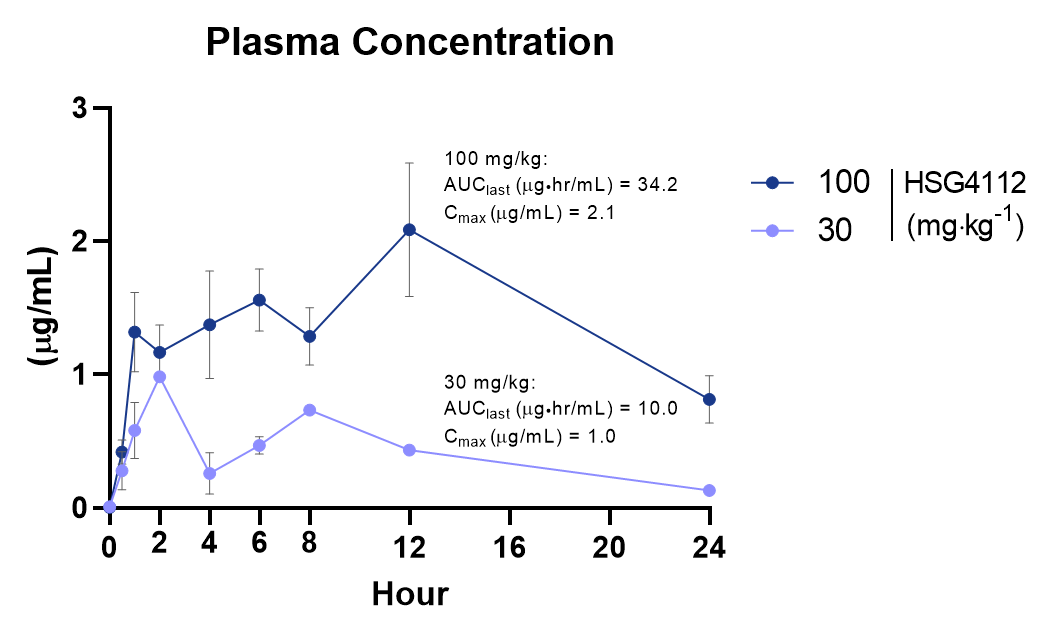


**Supplementary Fig. 2 Plasma concentration of HSG4112 in HFD-mice (n = 3 at each time point) after 6-week administration. Mean ± SEM.**


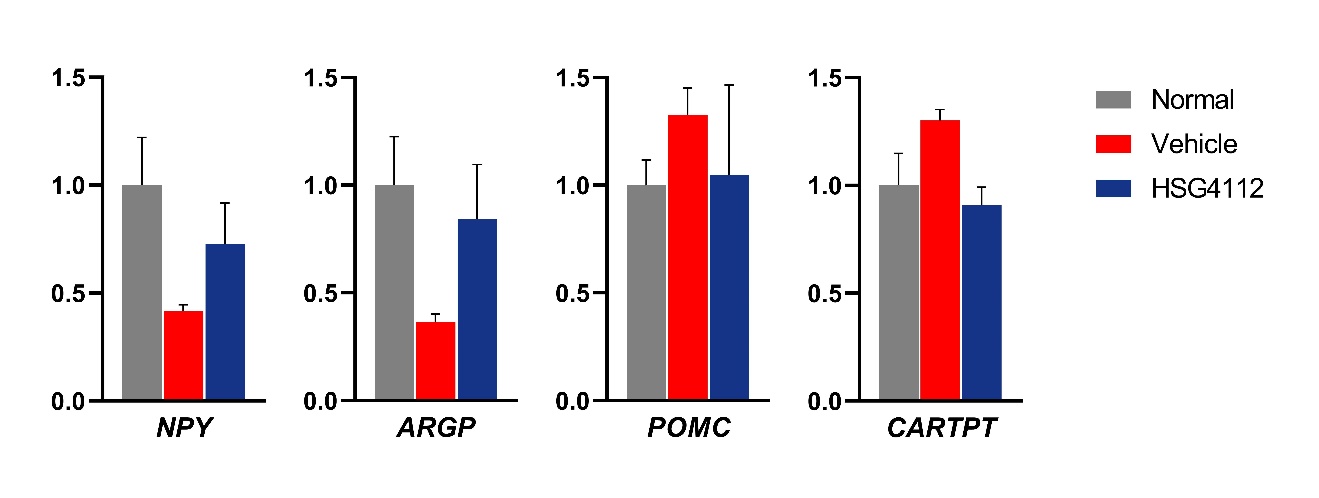


**Supplementary Fig. 3 Relative expression of genes related to leptin sensitivity and energy homeostasis in the hypothalamus. One-way ANOVA with Dunnett’s multiple comparison test. Not significant; (n = 4). Mean ± SEM.**


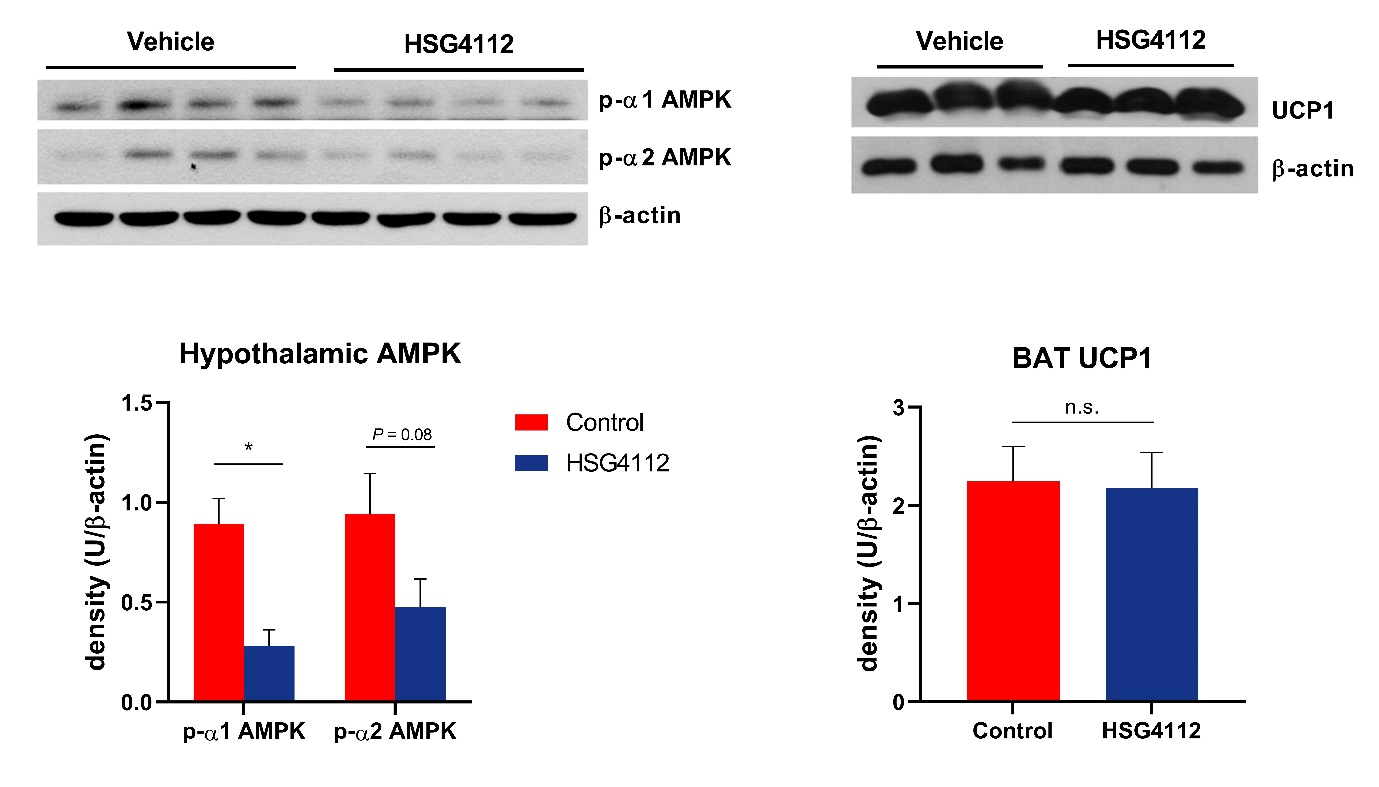


**Supplementary Fig. 4 For western blot assay, HFD-mice were treated for 11 days before sacrifice. Phosphorylated hypothalamic AMPK ɑ1 and ɑ2, and BAT UCP1, and their quantifications. Student’s t-test. **P* < 0.05. n.s. = not significant. Mean ± SEM.**
